# Risk of inflammatory bowel disease appears to vary across different frequency, amount, and subtype of alcoholic beverages

**DOI:** 10.3389/fnut.2022.918754

**Published:** 2022-07-27

**Authors:** Bi-Xia Liu, Jing Yang, Chunyan Zeng, Xi-Jian Dai, Youxiang Chen

**Affiliations:** ^1^Department of Gastroenterology, Digestive Disease Hospital, The First Affiliated Hospital of Nanchang University, Nanchang, China; ^2^Department of Radiology, The Second Affiliated Hospital of Nanchang University, Nanchang, China

**Keywords:** inflammatory bowel disease, Crohn's disease, ulcerative colitis, alcohol consumption, prospective cohort, risk factor, UK Biobank, alcoholic beverage

## Abstract

**Objective:**

Inflammatory bowel disease (IBD) and alcohol use has become a significant and growing public health concern. Alcohol use has been reported to be the most-avoided diet item among IBD patients. However, knowledge regarding the impact of different classes of alcoholic beverages on the management of IBD is limited. Our study aims to evaluate the association of different frequencies, amounts, and subtypes of alcoholic beverages with IBD risk.

**Methods:**

The UK Biobank comprised 7,095 subjects with IBD and 4,95,410 subjects without IBD. Multivariate Logistic regression, stratifying analysis, and interaction terms were used to estimate the odds ratios (ORs) and 95% confidence intervals (95% CIs) of IBD. A generalized additive model was used to evaluate the linearity associations of the total amount of all alcoholic beverages or that of each of five alcoholic beverages with IBD risk.

**Results:**

Compared with non-drinkers, the IBD risk was 12 to 16% lower in red wine consumers (1–2 glasses/week, OR [95%CI], 0.88 [0.80, 0.97]; 3–4 glasses/week, 0.84 [0.76, 0.93]; ≥5 glasses/week, 0.86 [0.78, 0.95]), whereas 12% higher in white wine and champagne consumers (1–2 glasses/week, 1.12 [1.03, 1.22]). Stratifying analysis showed low-frequency red wine consumers were associated with a lower IBD risk (0.85 [0.74, 0.97]), whereas spirits consumers were associated with a higher risk (1.28 [1.03, 1.59]). High doge of red wine consumers were associated with a lower IBD risk (above guidelines, 0.80 [0.67, 0.97]; double above, 0.83 [0.71, 0.97]), whereas high doge white wine and champagne (1.32 [1.09, 1.61]) and beer and cider (1.26 [1.02, 1.54]) consumers were associated with a higher IBD risk. White wine and champagne showed a significant interaction effect with high doge alcohol consumption (1.27 [1.03–1.58], *p* = 0.029). The dose-response association showed an increased IBD risk with more number of alcohol consumption of white wine and champagne, beer and cider, or the total amount of all alcoholic beverages. However, red wine is at low risk across the whole dose cycle.

**Conclusions:**

The IBD risk appears to vary across different frequencies, amounts, and subtypes of alcoholic beverages. Overall, alcohol intake is not recommended.

## Introduction

Crohn's disease (CD) and ulcerative colitis (UC) are collectively known as inflammatory bowel disease (IBD). IBD has become a significant and growing public health concern and has conveyed a high rate of morbidity and mortality (1, 2). IBD is progressive and immune-mediated inflammatory disease of the gastrointestinal tract. The established importance of environmental factors (e.g., diet and gut microenvironment) in the development of IBD has been widely reported (3, 4). Dietary triggers have aroused significant interest, as identifying modifiable dietary factors could help reduce relapse frequency, thereby limiting steroid use and decreasing hospitalizations (5). However, the exact etiological mechanism of IBD still remains unknown.

Alcohol use is a leading risk factor for disease burden worldwide, accounting for nearly 10% of global deaths among populations aged 15 to 49 years, and poses dire ramifications for future population health in the absence of policy action today (6). Alcohol use in patients with IBD is common (7), and even though its prevalence in patients with IBD appears to be similar to the general population, it has been reported to be the most-avoided diet item among IBD patients (8). Niccum et al. found that alcohol consumption is associated with an increased risk of microscopic colitis (9). Jowett et al. found that alcohol consumption is associated with a higher risk of relapse in patients with UC (10). Mantzouranis et al. reviewed several literature and found that alcohol consumption is associated with worse IBD symptoms among patients who consumed alcoholic beverages compared with those who did not consume alcoholic beverages (11). Patients with alcohol abuse disorder have a similar microbial signature to that of patients with IBD, and ethanol ingested from alcoholic beverages has been widely known to impair gut barrier permeability and function (1, 12–15), which may suggest that the alcohol consumption is involved in modulating the microbiome and facilitating intestinal inflammation, and therefore could facilitate IBD pathogenesis. However, beneficial effect on our health of some classes of alcoholic beverages have also been reported in recent years (16–18). However, knowledge regarding the impact of different classes of alcoholic beverages on the management of IBD are limited.

The observed relationships between consumption of alcoholic beverages and diseases are often non-linear, for example, with low-to-moderate alcohol consumption being protective and heavy alcohol consumption being harmful, or J-shaped risk of diseases with the amount of consumption of alcoholic beverages (18–22). There is limited high-quality epidemiologic evidence for the effect of different classes of alcoholic beverages on IBD. In the present large longitudinal observational study, we systematically investigated the associations of different classes of alcoholic beverages with IBD risk, as well as their associations among different frequencies and amounts of alcohol consumption. We further examined the “dose”-response association between alcohol consumption and IBD risk by drawing the risk trajectory of IBD with different amounts of alcohol consumption.

## Materials and methods

### Study population

The UK Biobank is a prospective cohort with a total of 5,02,505 subjects recruited from March 2006 to December 2010. We discovered 7,095 IBD cases, which consists of 2,027 cases who only have a diagnosis of Crohn's disease, 4,334 cases who only have a diagnosis of ulcerative colitis, and 734 cases who are both diagnosed with Crohn's disease and ulcerative colitis. Ethical approval of the UK Biobank was obtained from the National Health System Northwest Multicenter Research Ethics Committee (REC reference: 16/NW/0274).

### Exposure and outcomes

IBD was ascertained by the International Classification of Diseases, Version 10 (ICD-10), terms from the UK Biobank data field 4,1270, which included Crohn's disease (code K50) and ulcerative colitis (code K51).

To assess the frequency of alcohol consumption (Field ID 1558), subjects were asked to answer the question: About how often do you drink alcohol? Seven responses were given: prefer not to answer, never, special occasions only, one to three times a month, once or twice a week, three or four times a week, and daily or almost daily. The frequency of alcohol consumption was classified into three categories: (1) high frequency (≥3 times/week); (2) low frequency (<3 times/week); and (3) never/special occasions only (18).

The average weekly intake of red wine (ID 1568), champagne plus white wine (ID 1578), beer and cider (ID 1588), spirits (ID 1598), and fortified wine (ID 1608) were calculated, respectively. For example, to assess the weekly intake of red wine, subjects were asked to answer the question: In an average week, how many glasses of red wine would you drink? A response with an exact value (e.g., 2 glasses/week) should be given.

The total amount of alcohol consumption was quantified by summing the average weekly intake of red wine, champagne plus white wine, beer and cider, spirits, and fortified wine. As shown in our previous study (18), the weekly intake level of alcohol was converted into units for wines (1 standard glass = 2 units), beer and cider (1 pint =2 units), and spirits (1 shot = 1 unit), and was classified into four categories: (1) non-drinker, previous drinker, or special occasions only; (2) within recommended guidelines: <14 UK units/week; (3) above recommended guidelines: ≥14 units/week and <28 units/week; and (4) 2-fold or more above the recommended guidelines: ≥28 units/week.

### Confounding factors

A wide range of sociodemographic factors, lifestyle factors, sleep phenotypes, and comorbidities was considered as covariates to adjust for any potential confounding. The covariates of sociodemographic factors included age, sex, ethnicity (white and non-white), education level (college or above, others), body mass index [BMI≥30 and <30 kg/m (2)], current employment status, Townsend deprivation index, and overall health rating. Current employment status was classified into employed (including those in paid employment or self-employed) and unemployed (including those in retired, looking after home and/or family, unable to work because of sickness or disability, doing unpaid or voluntary work, or being full- or part-time students, and unemployed).

The covariates of lifestyle factors included smoking status (never, previous, or current) and usually walking pace (normal, slow, and fast walking pace). The covariates of comorbidities included cerebrovascular diseases, cardiovascular diseases, diabetes, respiratory disease, and cancer. The covariates of sleep phenotype included sleep duration (normal, short, and long sleep duration), early awakening, napping during the day, daytime dozing/sleeping (narcolepsy), sleeplessness or insomnia, and snoring.

### Statistical analyses

Continuous variables are presented as mean ± SD, and categorical variables are presented as a number (percentage). We used the unpaired *t*-test and χ^2^ test to compare differences between groups where appropriate.

We used Logistic regression analysis to estimate the odds ratios (ORs) and 95% confidence intervals (95% CIs). Multivariate Logistic regression analysis was used to evaluate the associations between consumption of alcoholic beverages and IBD risk after adjusting for sociodemographic factors, lifestyle factors, sleep phenotypes, comorbidities, frequency of alcohol consumption, amount of weekly intake level of alcohol consumption, and alcoholic beverages. Stratifying analysis was further used to examine the association of different subtypes of alcoholic beverages with the risk of IBD, separated by frequency of alcohol consumption and the total amount of alcohol consumption, respectively.

Interaction terms were employed for the overall sample to explore potential interactions between different subtypes of alcoholic beverages on the risk of IBD in the final model. A generalized additive model was used to evaluate the linearity associations of the total amount of alcohol consumption or that of alcoholic beverages with the risk of IBD.

All analyses were conducted with SPSS version 24.0 and R Statistical Software version 4.0. A two-tailed *p*-value <0.05 was considered significant.

## Results

### Sample characteristics

The demographic characteristics of the study population are presented in Table 1. Compared with subjects without IBD, those with IBD had a poor socioeconomic status [a lower education level (*p* < 0.001), a poor employment status (*p* < 0.001), and a higher Townsend deprivation score (*p* < 0.001)], a poor overall health rating (*p* < 0.001), more sleep disorders (long and short sleep duration; *p* < 0.001, usually napping during the day, *p* < 0.001; narcolepsy, *p* < 0.001; sleeplessness, *p* < 0.001; snoring, *p* < 0.001), and more comorbidities (cerebrovascular diseases, *p* < 0.001; cardiovascular diseases, *p* < 0.001; diabetes, *p* < 0.001; respiratory disease, *p* < 0.001; and cancer, *p* < 0.001). Furthermore, they were more likely to be males (*p* < 0.001), white ethnicity (*p* = 0.004), smokers (*p* < 0.001), and slow walking pacers (*p* < 0.001), but they were less likely to be frequent alcohol drinkers (*p* < 0.001), heavy drinkers (*p* < 0.001), red wine drinkers (*p* < 0.001), white wine and champagne drinkers (*p* < 0.001), and beer and cider drinkers (*p* < 0.001), whereas they were more likely to be spirits drinkers (*p* < 0.001) and fortified wine drinkers (*p* < 0.001).

**Table 1:** Characteristics of UK Biobank cohort.

**Characteristics**	**IBD related risk**		***p*** **-value**
	**IBD(*****n*** = **7095)**	**Non-IBD(*****n*** = **495,410)**	
Age to 2021 (years), mean ± SD	70.2 ± 8.0	69.5 ± 8.1	<0.001
Age categories, *N* (%)			<0.001
≤ 60 years	1,105 (15.6)	89,836 (18.1)	
61-70 years	2,076 (29.3)	154,325 (31.2)	
≥71 years	3,914 (55.2)	251,248 (50.7)	
Sex (male), *N* (%)	3,482 (49.1)	225,640 (45.5)	<0.001
Education (college or above), *N* (%)	1,827 (26.3)	159,336 (32.8)	<0.001
Ethnicity (white), *N* (%)	6,718 (95.3)	465,977 (94.5)	0.004
Obesity (BMI ≥30 kg/m [2]), *N* (%)	1,735 (24.6)	120,512 (24.5)	0.76
Overall health rating, *N* (%)			<0.001
Excellent or good	3,813 (54.1)	367,109 (74.6)	
Fair or poor	3,230 (45.9)	124,995 (25.4)	
Employment status (in paid), *N* (%)	3,579 (50.8)	283,570 (57.6)	<0.001
Smoking (previous or current), *N* (%)	3,881 (55.0)	222,153 (45.1)	<0.001
Townsend deprivation, mean ± SD	−1.1 ± 3.2	−1.3 ± 3.1	<0.001
Usually walking pace, *N* (%)			<0.001
Normal	3,720 (53.3)	259,126 (52.8)	
Slow	897 (12.9)	40,070 (8.2)	
Fast	2,361 (33.8)	191,770 (39.1)	
Sleep duration, mean ± SD			
Sleep duration categories, *N* (%)			<0.001
Short sleep duration (≤ 6 h)	1,898 (27.1)	121,354 (24.7)	
Normal sleep duration (7-8 h)	4,501 (64.2)	332,189 (67.6)	
Long sleep duration (≥9 h)	609 (8.7)	37,738 (7.7)	
Early awakening, *N* (%)			<0.001
Not very easy/Not at all easy	1,525 (21.8)	88,173 (18.0)	
Fairly easy	3,363 (48.0)	243,283 (49.7)	
Very easy	2,119 (30.2)	158,368 (32.3)	
Nap during day, *N* (%)			<0.001
Never/rarely	3,544 (50.1)	277,525 (56.2)	
Sometimes	3,036 (42.9)	189,619 (38.4)	
Usually	493 (7.0)	26,393 (5.3)	
Daytime dozing/sleeping (narcolepsy), N (%)			<0.001
Never/rarely	5,064 (72.0)	373,642 (76.0)	
Sometimes	1,693 (24.1)	104,276 (21.2)	
Often/ All of the time	281 (4.0)	13,814 (2.8)	
Sleeplessness or insomnia, *N* (%)			<0.001
Never/rarely	1,473 (20.8)	119,302 (24.2)	
Sometimes	3,182 (45.0)	235,655 (47.7)	
Usually	2,420 (34.2)	138,969 (28.1)	
Snoring, *N* (%)	4,177 (64.0)	287,915 (62.7)	0.03
Cerebrovascular diseases, *N* (%)	282 (4.0)	12,205 (2.5)	<0.001
Cardiovascular diseases, *N* (%)	858 (12.1)	37,528 (7.6)	<0.001
Diabetes, *N* (%)	794 (11.2)	30,496 (6.2)	<0.001
Respiratory disease, *N* (%)	2,239 (31.6)	88,521 (17.9)	<0.001
Cancer, *N* (%)	1,369 (19.3)	74,119 (15.0)	<0.001
Frequency of alcohol intake, *N* (%)			<0.001
Never and special occasions only	1,748 (24.7)	96,901 (19.6)	
Once a month-twice a week	2,559 (36.2)	182,588 (37.0)	
≥3 times a week	2,763 (39.1)	214,444 (43.4)	
Alcohol consumption (dosage), UK units/week, mean ± SD	13.5 ± 17.8	14.8 ± 18.1	<0.001
Alcohol consumption, dosage, *N* (%)			<0.001
Never drinker, previous-drinkers or special occasions only	2,623 (37.0)	152,651 (30.8)	
Within guidelines	1,800 (25.4)	137,247 (27.7)	
Above guidelines	1,512 (21.3)	115,304 (23.3)	
Double above the guidelines or more	1,160 (16.3)	90,208 (18.2)	
Red wine drinkers, *N* (%)			<0.001
Non-drinkersNon-drinkers	4,391 (61.9)	268,247 (54.1)	
1–2 glasses/week	866 (12.2)	70,495 (14.2)	
3–4 glasses/week	627 (8.8)	54,219 (10.9)	
≥5 glasses/week	1,211 (17.1)	102,449 (20.7)	
White wine and champagne drinkers			<0.001
Non-drinkers	4,735 (66.7)	307,747 (62.1)	
1–2 glasses/week	1,052 (14.8)	80,226 (16.2)	
3–4 glasses/week	537 (7.6)	44,041 (8.9)	
≥5 glasses/week	771 (10.9)	63,396 (12.8)	
Beer and cider drinkers, *N* (%)			<0.001
Non-drinkers	4,639 (65.4)	314,004 (63.4)	
1–2 glasses/week	952 (13.4)	75,681 (15.3)	
3–4 glasses/week	485 (6.8)	35,459 (7.2)	
≥5 glasses/week	1,019 (14.4)	70,266 (14.2)	
Spirits drinkers, *N* (%)			0.001
Non-drinkers	5,327 (75.1)	366,804 (74.0)	
1–2 glasses/week	821 (11.6)	65,500 (13.2)	
3–4 glasses/week	389 (5.5)	25,957 (5.2)	
≥5 glasses/week	558 (7.9)	37,149 (7.5)	
Fortified wine drinkers, *N* (%)			0.13
Non-drinkers	6,646 (93.7)	461,067 (93.1)	
1–2 glasses/week	329 (4.6)	26,092 (5.3)	
3–4 glasses/week	66 (0.9)	4,583 (0.9)	
≥5 glasses/week	54 (0.8)	3,668 (0.7)	

### Association between different alcoholic beverages and IBD risk

The associations of different alcoholic beverages with the risk of IBD are shown in Table 2. In the final multivariate model, compared with non-drinkers, the risk of IBD was 12 to 16% lower in red wine consumers (1–2 glasses/week, OR [95%CI], 0.88 [0.80, 0.97]; 3–4 glasses/week, 0.84 [0.76, 0.93]; ≥5 glasses/week, 0.86 [0.78, 0.95]) regardless of the amount of red wine, whereas 12% higher in white wine and champagne consumers of 1–2 glasses/week (1.12 [1.03, 1.22]). Furthermore, the higher risk was not significant when the amount of white wine and champagne was higher (3–4 glasses/week, 1.04 [0.94, 1.16]; ≥5 glasses/week, 1.04 [0.93, 1.15]). Compared with non-drinkers, beer and cider consumers, spirits wine consumers, and fortified wine consumers were not significantly associated with IBD risk regardless of the amount of alcohol.

**Table 2:** Association of different subtypes of alcoholic beverages with IBD risk.

**Variables**	**Model 1**		**Model 2**		**Model 3**		**Model 4**	
	**OR (95%Cl)**	***p*** **-value**	**OR (95%Cl)**	***p*** **-value**	**OR (95%Cl)**	***p*** **-value**	**OR (95%Cl)**	***p*** **-value**
Red wine drinkers								
Non-drinkers	Reference		Reference		Reference		Reference	
1–2 glasses/week	0.86 (0.79–0.92)	<0.001	0.86 (0.80–0.93)	<0.001	0.87 (0.81–0.95)	0.001	0.88 (0.80–0.97)	0.007
3–4 glasses/week	0.82 (0.75–0.89)	<0.001	0.80 (0.73–0.88)	<0.001	0.82 (0.75–0.90)	<0.001	0.84 (0.76–0.93)	0.001
≥5 glasses/week	0.82 (0.76–0.87)	<0.001	0.79 (0.74–0.85)	<0.001	0.81 (0.76–0.87)	<0.001	0.86 (0.78–0.95)	0.004
White wine and champagne drinkers
Non-drinkers	Reference		Reference		Reference		Reference	
1–2 glasses/week	0.98 (0.91–1.05)	0.47	1.00 (0.93–1.07)	0.92	1.01 (0.94–1.09)	0.80	1.12 (1.03–1.22)	0.008
3–4 glasses/week	0.93 (0.85–1.02)	0.13	0.92 (0.83–1.01)	0.08	0.93 (0.85–1.03)	0.17	1.04 (0.94–1.16)	0.47
≥5 glasses/week	0.93 (0.86–1.01)	0.07	0.91 (0.84–0.99)	0.026	0.93 (0.86–1.01)	0.08	1.04 (0.93–1.15)	0.51
Beer and cider drinkers								
Non-drinkers	Reference		Reference		Reference		Reference	
1–2 glasses/week	0.88 (0.82–0.95)	0.001	0.89 (0.82–0.96)	0.002	0.90 (0.83–0.97)	0.006	0.97 (0.89–1.05)	0.42
3–4 glasses/week	0.89 (0.81–0.98)	0.021	0.88 (0.80–0.98)	0.019	0.89 (0.81–0.99)	0.035	0.97 (0.86–1.08)	0.54
≥5 glasses/week	0.83 (0.77–0.90)	<0.001	0.83 (0.76–0.90)	<0.001	0.84 (0.77–0.91)	<0.001	0.92 (0.82–1.03)	0.16
Spirits drinkers								
Non-drinkers	Reference		Reference		Reference		Reference	
1–2 glasses/week	0.92 (0.85–0.99)	0.025	0.92 (0.85–0.99)	0.036	0.93 (0.86–1.01)	0.07	1.00 (0.92–1.08)	0.91
3–4 glasses/week	1.02 (0.92–1.14)	0.66	0.99 (0.88–1.11)	0.83	0.99 (0.89–1.11)	0.88	1.07 (0.95–1.20)	0.25
≥5 glasses/week	0.96 (0.88–1.05)	0.36	0.94 (0.86–1.04)	0.23	0.94 (0.86–1.03)	0.21	1.03 (0.93–1.14)	0.60
Fortified wine drinkers								
Non-drinkers	Reference		Reference		Reference		Reference	
1–2 glasses/week	0.95 (0.85–1.06)	0.37	0.96 (0.85–1.08)	0.52	0.97 (0.87–1.10)	0.67	1.03 (0.91–1.16)	0.64
3–4 glasses/week	1.04 (0.81–1.34)	0.75	0.95 (0.73–1.25)	0.73	0.96 (0.73–1.26)	0.78	1.02 (0.78–1.34)	0.89
≥5 glasses/week	1.01 (0.77–1.33)	0.93	1.02 (0.76–1.35)	0.92	1.02 (0.76–1.35)	0.92	1.10 (0.82–1.47)	0.53

*Model 1 analyses were adjusted for age, sex, education, ethnicity, employment, and overall health rating.*

*Model 2 analyses were further adjusted for smoking, Townsend, BMI, usually walking pace, sleep duration, getting up easily in the morning, nap during the day, daytime dozing/sleeping, sleeplessness, and snoring.*

*Model 3 analyses were further adjusted for cardiovascular diseases, diabetes, respiratory disease, and cancer.*

*Model 4 Analyses were further adjusted for frequency of alcohol intake, amount of weekly intake level of alcohol consumption, red wine, champagne plus white wine, beer and cider, spirits, and fortified wine*.

### Sensitivity analysis of the association between alcoholic beverages and IBD risk

We further analyzed the association of alcoholic beverages with IBD risk among those subjects who only consumed one alcoholic beverage (Table 3). Compared with subjects without IBD, those subjects with IBD were less likely to be red wine drinkers (*p* < 0.001) and white wine and champagne drinkers (*p* < 0.001). However, no significant differences were found in beer and cider drinkers (*p* = 0.1), spirits drinkers (*p* = 0.49), and fortified wine drinkers (*p* = 0.89) between subjects with and without IBD. In the final multivariate model, compared with non-drinkers, the risk of IBD was 24% lower in red wine consumers of 3–4 glasses/week (0.76 [0.59, 1.00]) and 23% lower in ≥5 glasses/week (0.77 [0.66, 0.91]). However, white wine and champagne drinkers were not significantly associated with the risk of IBD.

**Table 3:** Characteristics of those subjects who only consumed one alcoholic beverage.

**Characteristics**	**IBD related risk**		***p*** **-value**
	**IBD(*****n*** = **7,095)**	**Non-IBD(*****n*** = **495,410)**	
Only red wine drinkers, *N* (%)			<0.001
Non-drinkers	2,623 (89.1)	152,651 (84.5)	
1–2 glasses/week	59 (2.0)	4,980 (2.8)	
3–4 glasses/week	66 (2.2)	5,904 (3.3)	
≥5 glasses/week	195 (6.6)	17,077 (9.5)	
Only white wine and champagne drinkers			0.001
Non-drinkers	2,623 (89.5)	152,651 (87.0)	
1–2 glasses/week	53 (1.8)	4,304 (2.5)	
3–4 glasses/week	57 (1.9)	4,551 (2.6)	
≥5 glasses/week	199 (6.8)	14,022 (8.0)	
Only beer and cider drinkers, *N* (%)			0.1
Non-drinkers	2,623 (83.9)	152,651 (82.5)	
1–2 glasses/week	81 (2.6)	5,738 (3.1)	
3–4 glasses/week	80 (2.6)	5,662 (3.1)	
≥5 glasses/week	344 (11.0)	21,039 (11.4)	
Only spirits drinkers, *N* (%)			0.49
Non-drinkers	2,623 (94.4)	152,651 (94.7)	
1–2 glasses/week	27 (1.0)	1,669 (1.0)	
3–4 glasses/week	38 (1.4)	1,733 (1.1)	
≥5 glasses/week	91 (3.3)	5,087 (3.2)	
Only fortified wine drinkers, *N* (%)			0.89
Non-drinkers	2,623 (99.4)	152,651 (99.3)	
1–2 glasses/week	6 (0.2)	456 (0.3)	
3–4 glasses/week	4 (0.2)	272 (0.2)	
≥5 glasses/week	6 (0.2)	397 (0.3)	

^#^0.05 ≤ p <0.1, *p <0.05, **p ≤ 0.01, and ***p ≤ 0.001 indicated p-values between IBD and non-IBD in male participants or female participants.

### Stratification analysis

We further examined the association of different alcoholic beverages with IBD risk, separated by frequency of alcohol intake (Table 4), amount of alcohol consumption (Table 5), and overall health rating (Table 6), respectively. In the final multivariate model, sociodemographic factors, lifestyle factors, sleep phenotypes, comorbidities, frequency of alcohol consumption, amount of weekly intake level of alcohol consumption, and alcoholic beverages were adjusted.

**Table 4:** Odds ratios and 95% CIs for the association between different alcoholic beverages and IBD risk, separated by frequency of alcohol intake.

**Variables (a)**	**Frequency of alcohol intake, OR (95%Cl)**			
	**Once a month-twice/week**	***p***	**≥3 times/week**	***p***
Red wine drinkers				
Non-drinkers	Reference		Reference	
1–2 glasses/week	0.85 (0.74–0.97)	0.015	0.95 (0.83–1.09)	0.46
3–4 glasses/week	0.89 (0.76–1.05)	0.18	0.83 (0.73–0.96)	0.009
≥5 glasses/week	0.89 (0.70–1.12)	0.31	0.88 (0.78–0.98)	0.023
White wine and champagne drinkers				
Non-drinkers	Reference		Reference	
1–2 glasses/week	1.08 (0.96–1.23)	0.21	1.18 (1.06–1.33)	0.004
3–4 glasses/week	1.11 (0.92–1.33)	0.28	1.04 (0.91–1.19)	0.60
≥5 glasses/week	1.11 (0.86–1.43)	0.42	1.05 (0.94–1.19)	0.39
Beer and cider drinkers				
Non-drinkers	Reference		Reference	
1–2 glasses/week	0.93 (0.81–1.07)	0.32	0.99 (0.88–1.11)	0.82
3–4 glasses/week	0.97 (0.80–1.17)	0.75	0.94 (0.81–1.10)	0.44
≥5 glasses/week	0.93 (0.73–1.17)	0.52	0.90 (0.78–1.04)	0.15
Spirits drinkers				
Non-drinkers	Reference		Reference	
1–2 glasses/week	1.01 (0.88–1.15)	0.94	0.98 (0.87–1.09)	0.67
3–4 glasses/week	1.08 (0.89–1.33)	0.44	1.04 (0.90–1.20)	0.62
≥5 glasses/week	1.28 (1.03–1.59)	0.029	0.94 (0.84–1.06)	0.31
Fortified wine drinkers				
Non-drinkers	Reference		Reference	
1–2 glasses/week	1.08 (0.88–1.32)	0.46	1.02 (0.88–1.19)	0.81
3–4 glasses/week	1.27 (0.78–2.08)	0.34	0.95 (0.68–1.32)	0.75
≥5 glasses/week	0.19 (0.03–1.38)	0.10	1.24 (0.92–1.66)	0.16

*Logistic regression analysis adjusted for age, sex, education, ethnicity, employment, overall health rating, smoking, Townsend, BMI, usually walking pace, sleep duration, getting up easily in the morning, nap during the day, daytime dozing/sleeping, sleeplessness, snoring, cardiovascular diseases, diabetes, respiratory disease, cancer, amount of weekly intake level of alcohol consumption, red wine, champagne plus white wine, beer and cider, spirits, and fortified wine*.

**Table 5:** Odds ratios and 95% CIs for the association between different alcoholic beverages and IBD risk, separated by the amount of alcohol consumption.

**Variables (b)**	**Alcohol consumption, dosage**					
	**Within guidelines**	***p*** **-value**	**Above guidelines**	***p-*** **value**	**Double above the guidelines or more**	***p*** **-value**
Red wine drinkers						
Non-drinkers	Reference		Reference		Reference	
1–2 glasses/week	0.89 (0.78–1.01)	0.08	0.89 (0.75–1.07)	0.21	1.02 (0.80–1.29)	0.90
3–4 glasses/week	0.91 (0.77–1.08)	0.27	0.80 (0.67–0.97)	0.021	0.86 (0.67–1.12)	0.27
≥5 glasses/week	0.97 (0.74–1.27)	0.83	0.88 (0.73–1.06)	0.17	0.83 (0.71–0.97)	0.02
White wine and champagne drinkers						
Non-drinkers	Reference		Reference		Reference	
1–2 glasses/week	1.11 (0.98–1.26)	0.09	1.12 (0.96–1.31)	0.14	1.32 (1.09–1.61)	0.005
3–4 glasses/week	1.11 (0.92–1.34)	0.27	1.12 (0.94–1.34)	0.21	0.93 (0.73–1.19)	0.57
≥5 glasses/week	1.31 (0.99–1.72)	0.06	1.04 (0.85–1.27)	0.70	1.02 (0.87–1.19)	0.85
Beer and cider drinkers						
Non-drinkers	Reference		Reference		Reference	
1–2 glasses/week	0.92 (0.81–1.06)	0.25	0.88 (0.75–1.03)	0.11	1.26 (1.02–1.54)	0.03
3–4 glasses/week	0.93 (0.76–1.14)	0.50	0.87 (0.71–1.06)	0.17	1.19 (0.93–1.52)	0.17
≥5 glasses/week	0.82 (0.62–1.10)	0.18	0.84 (0.67–1.05)	0.12	1.07 (0.88–1.31)	0.48
Spirits drinkers						
Non-drinkers	Reference		Reference		Reference	
1–2 glasses/week	1.05 (0.93–1.20)	0.41	1.02 (0.89–1.17)	0.78	0.82 (0.67–1.00)	0.051
3–4 glasses/week	1.06 (0.86–1.30)	0.59	0.99 (0.81–1.20)	0.92	1.16 (0.94–1.43)	0.17
≥5 glasses/week	1.26 (1.00–1.58)	0.053	1.05 (0.87–1.26)	0.62	0.86 (0.73–1.02)	0.08
Fortified wine drinkers						
Non-drinkers	Reference		Reference		Reference	
1–2 glasses/week	1.03 (0.85–1.26)	0.75	1.10 (0.91–1.34)	0.32	0.99 (0.77–1.28)	0.93
3–4 glasses/week	1.32 (0.78–2.22)	0.30	1.01 (0.66–1.52)	0.98	0.89 (0.52–1.51)	0.66
≥5 glasses/week	0.45 (0.06–3.23)	0.43	1.25 (0.79–1.97)	0.34	1.08 (0.73–1.60)	0.71

*Logistic regression analysis adjusted for age, sex, education, ethnicity, employment, overall health rating, smoking, Townsend, BMI, usually walking pace, sleep duration, getting up easily in the morning, nap during the day, daytime dozing/sleeping, sleeplessness, snoring, cardiovascular diseases, diabetes, respiratory disease, cancer, frequency of alcohol intake, red wine, champagne plus white wine, beer and cider, spirits, and fortified wine*.

**Table 6:** Odds ratios and 95% CIs for the association between different alcoholic beverages and IBD risk, separated by overall health rating.

**Variables**	**Overall health rating, OR (95%Cl)**			
	**Excellent or good**	***p***	**Fair or poor**	***p***
Red wine drinkers				
Non-drinkers	Reference		Reference	
1–2 glasses/week	0.89 (0.79–1.00)	0.044	0.85 (0.73–0.99)	0.039
3–4 glasses/week	0.77 (0.67–0.87)	<0.001	0.98 (0.83–1.15)	0.77
≥5 glasses/week	0.84 (0.74–0.95)	0.006	0.88 (0.75–1.03)	0.12
White wine and champagne drinkers				
Non-drinkers	Reference		Reference	
1–2 glasses/week	1.12 (1.01–1.24)	0.035	1.12 (0.98–1.28)	0.11
3–4 glasses/week	1.11 (0.98–1.27)	0.11	0.91 (0.75–1.10)	0.33
≥5 glasses/week	1.04 (0.91–1.19)	0.56	1.03 (0.86–1.22)	0.78
Beer and cider drinkers				
Non-drinkers	Reference		Reference	
1–2 glasses/week	0.96 (0.86–1.07)	0.45	0.97 (0.84–1.11)	0.62
3–4 glasses/week	0.99 (0.86–1.14)	0.87	0.92 (0.77–1.10)	0.36
≥5 glasses/week	0.94 (0.81–1.09)	0.43	0.89 (0.74–1.06)	0.19
Spirits drinkers				
Non-drinkers	Reference		Reference	
1–2 glasses/week	0.98 (0.88–1.09)	0.70	1.01 (0.88–1.17)	0.85
3–4 glasses/week	1.07 (0.92–1.24)	0.38	1.06 (0.88–1.28)	0.53
≥5 glasses/week	1.01 (0.88–1.16)	0.92	1.05 (0.90–1.23)	0.56
Fortified wine drinkers				
Non-drinkers	Reference		Reference	
1–2 glasses/week	0.99 (0.85–1.16)	0.91	1.11 (0.91–1.36)	0.32
3–4 glasses/week	1.03 (0.73–1.45)	0.87	1.01 (0.64–1.60)	0.97
≥5 glasses/week	1.30 (0.92–1.84)	0.14	0.79 (0.46–1.35)	0.38

*Logistic regression analysis adjusted for age, sex, education, ethnicity, employment, overall health rating, smoking, Townsend, BMI, usually walking pace, sleep duration, getting up easily in the morning, nap during the day, daytime dozing/sleeping, sleeplessness, snoring, cardiovascular diseases, diabetes, respiratory disease, cancer, amount of weekly intake level of alcohol consumption, red wine, champagne plus white wine, beer and cider, spirits, and fortified wine*.

When stratifying our analysis by frequency of alcohol intake (Table 4), we found that, among the subjects who usually reported consumption of alcohol at a low frequency, consumption of red wine was associated with a lower risk of IBD (1–2 glasses/week, 0.85 [0.74, 0.97]), whereas consumption of spirits was associated with a higher risk of IBD (≥5 glasses/week, 1.28 [1.03, 1.59]). Among the subjects who usually reported consumption of alcohol at a high frequency, consumption of red wine was still associated with a lower risk of IBD (3–4 glasses/week, 0.83 [0.73, 0.96]; ≥5 glasses/week, 0.88 [0.78, 0.98]), whereas consumption of white wine and champagne was associated with a higher risk of IBD (1–2 glasses/week, 1.18 [1.06, 1.33]).

When stratifying our analysis by the amount of alcohol consumption (Table 5), we found that, among those subjects who usually reported consumption of alcohol within guidelines, all alcoholic beverages were not associated with IBD risk. Among those subjects who usually reported consumption of alcohol above guidelines, consumption of red wine was associated with a lower risk of IBD (3–4 glasses/week, 0.80 [0.67, 0.97]). Among those subjects who usually reported consumption of alcohol double above the guidelines, consumption of red wine was still associated with a lower risk of IBD (≥5 glasses/week, 0.83 [0.71, 0.97]), whereas consumption of white wine and champagne (1–2 glasses/week, 1.32 [1.09, 1.61]) and consumption of beer and cider (1–2 glasses/week,1.26 [1.02, 1.54]) were associated with a higher risk of IBD, respectively.

We also examined the association of different alcoholic beverages with IBD risk when stratifying our analysis by overall health rating (Table 6). Among those subjects with excellent or good overall health rating, consumption of red wine was associated with 11% to 23% lower risks of IBD (1–2 glasses/week, 0.89 [0.79, 1.00]; 3–4 glasses/week, 0.77 [0.67, 0.87]; ≥5 glasses/week, 0.84 [0.74, 0.95]) regardless of the amount of red wine, whereas consumption of white wine and champagne (1–2 glasses/week, 1.12 [1.01, 1.24]) was associated with a 12% higher risk of IBD. For those subjects with poor overall health rating, only consumption of 1–2 glasses/week of red wine was associated with a 15% lower risk of IBD (1–2 glasses/week, 0.85 [0.73, 0.99]), whereas the higher risk was not significant among white wine and champagne consumers. Other alcoholic beverages were not associated with IBD risk among those subjects with both good and poor overall health ratings. Therefore, our study suggests that the association between alcoholic beverages and the risk of IBD are not affected by overall health rating.

### Interaction effects on IBD risk

Tables 7–11 showed that there were significant interaction effects between different alcoholic beverages, overall health rating, and amount of alcohol consumption (alcoholic beverage × alcoholic beverage, alcoholic beverage × overall health rating, alcoholic beverage × amount of alcohol consumption) regarding the risk of IBD after adjusting for sociodemographic factors, lifestyle factors, sleep phenotypes, comorbidities, frequency of alcohol consumption, amount of alcohol consumption, and alcoholic beverages.

**Table 7:** Odds ratios and 95% CIs for the interaction effect between red wine and risk of IBD.

	**Red wine drinkers**						
	**Non-drinkers**	**1–2 glasses/week**	**p**	**3–4 glasses/week**	***p***	**≥5 glasses/week**	***p***
Overall health rating							
Excellent or good	Reference	Reference		Reference		Reference	
Fair or poor	Reference	0.92 (0.78–1.09)	0.32	1.20 (1.00–1.44)	0.06	0.98 (0.85–1.13)	0.79
Alcohol consumption, dosage							
Never drinker, previous–drinkers or special occasions only	Reference	Reference		Reference		Reference	
Within guidelines	Reference	N/A		N/A		N/A	
Above guidelines	Reference	1.06 (0.87–1.30)	0.56	0.95 (0.77–1.17)	0.63	0.96 (0.74–1.25)	0.75
Double above the guidelines or more	Reference	1.26 (0.97–1.63)	0.08	1.01 (0.76–1.35)	0.93	0.92 (0.71–1.20)	0.54
White wine and champagne drinkers							
Non-drinkers	Reference	Reference		Reference		Reference	
1–2 glasses/week	Reference	1.21 (0.98–1.49)	0.08	0.93 (0.72–1.19)	0.55	1.16 (0.95–1.43)	0.15
3–4 glasses/week	Reference	1.31 (0.96–1.78)	0.09	1.05 (0.80–1.38)	0.73	1.25 (0.96–1.62)	0.09
≥5 glasses/week	Reference	1.20 (0.92–1.56)	0.18	1.02 (0.75–1.38)	0.91	1.02 (0.84–1.25)	0.82
Beer and cider drinkers							
Non-drinkers	Reference	Reference		Reference		Reference	
1–2 glasses/week	Reference	1.17 (0.95–1.45)	0.14	1.16 (0.91–1.47)	0.24	1.16 (0.95–1.41)	0.15
3–4 glasses/week	Reference	1.12 (0.84–1.50)	0.45	1.11 (0.80–1.52)	0.54	1.13 (0.89–1.45)	0.32
≥5 glasses/week	Reference	1.10 (0.88–1.39)	0.40	1.15 (0.88–1.49)	0.31	1.14 (0.95–1.38)	0.17
Spirits drinkers							
Non-drinkers	Reference	Reference		Reference		Reference	
1–2 glasses/week	Reference	1.16 (0.93–1.45)	0.18	1.12 (0.88–1.44)	0.36	1.07 (0.87–1.32)	0.53
3–4 glasses/week	Reference	1.20 (0.86–1.68)	0.29	1.31 (0.93–1.84)	0.12	1.13 (0.86–1.49)	0.39
≥5 glasses/week	Reference	1.22 (0.92–1.62)	0.18	1.34 (0.99–1.82)	0.06	0.86 (0.68–1.08)	0.19
Fortified wine drinkers							
Non-drinkers	Reference	Reference		Reference		Reference	
1–2 glasses/week	Reference	1.38 (1.00–1.92)	0.05	1.10 (0.76–1.59)	0.63	1.18 (0.85–1.64)	0.32
3–4 glasses/week	Reference	0.77 (0.35–1.69)	0.51	0.96 (0.45–2.04)	0.91	0.73 (0.36–1.48)	0.38
≥5 glasses/week	Reference	1.70 (0.78–3.72)	0.19	1.46 (0.57–3.71)	0.43	1.25 (0.62–2.53)	0.54

*Logistic regression analysis adjusted for age, sex, education, ethnicity, employment, overall health rating, smoking, Townsend, BMI, usually walking pace, sleep duration, getting up easily in the morning, nap during the day, daytime dozing/sleeping, sleeplessness, snoring, cardiovascular diseases, diabetes, respiratory disease, cancer, frequency of alcohol intake, red wine, champagne plus white wine, beer and cider, spirits, and fortified wine*.

There is an approximate interaction effect between 1–2 glasses/week of red wine and 1–2 glasses/week of fortified wine (1.38 [1.00–1.92], *p* = 0.05). However, other interaction effects of red wine with overall health rating, amount of alcohol consumption, white wine and champagne, beer and cider, or spirits on IBD risk were not found (Table 7). Consumption of 1–2 glasses/week of white wine and champagne showed significant interaction effects with double above the guidelines of alcohol consumption (1.27 [1.03–1.58], *p* = 0.029) and more than five glasses/week of beer and cider (1.46 [1.20–1.79], *p* < 0.001) on a higher risk of IBD, respectively (Table 8). A similar interaction pattern was observed between 3–4 glasses/week of white wine and champagne and 3–4 glasses/week of beer and cider (1.45 [1.05–2.01], p = 0.024). Several interaction effects between beer and cider and poor health rating (3–4 glasses/week × poor health, *p* = 0.04; ≥5 glasses/week × poor health, *p* = 0.001) or spirits (1–2 glasses/week beer and cider ×3–4 glasses/week spirits, *p* = 0.026; 1–2 glasses/week × ≥5 glasses/week, *p* = 0.003; 3-4 glasses/week ×1–2 glasses/week, *p* = 0.023) on higher risks of IBD were observed (Table 9). There is an interaction effect between ≥5 glasses/week of spirits and 3–4 glasses/week of fortified wine (*p* = 0.032) (Table 10). However, this interaction pattern was not observed between fortified wine and overall health rating and between fortified wine and amount of alcohol consumption, respectively (Table 11).

**Table 8:** Odds ratios and 95% CIs for the interaction effect between white wine and champagne and the risk of IBD.

	**White wine and champagne drinkers**						
	**Non-drinkers**	**1–2 glasses/week**	***p***	**3–4 glasses/week**	***p***	**≥5 glasses/week**	***p***
Overall health rating							
Excellent or good	Reference	Reference		Reference		Reference	
Fair or poor	Reference	0.99 (0.86–1.15)	0.94	0.86 (0.70–1.06)	0.16	0.97 (0.82–1.15)	0.69
Alcohol consumption, dosage							
Never drinker, previous–drinkers or special occasions only	Reference	Reference		Reference		Reference	
Within guidelines	Reference	N/A		N/A		N/A	
Above guidelines	Reference	1.04 (0.87–1.25)	0.64	1.00 (0.79–1.25)	0.97	0.82 (0.62–1.08)	0.15
Double above the guidelines or more	Reference	1.27 (1.03–1.58)	0.029	0.86 (0.65–1.14)	0.30	0.78 (0.59–1.02)	0.07
Beer and cider drinkers							
Non-drinkers	Reference	Reference		Reference		Reference	
1–2 glasses/week	Reference	1.17 (0.97–1.41)	0.11	1.26 (0.98–1.61)	0.07	1.14 (0.91–1.42)	0.26
3–4 glasses/week	Reference	1.27 (0.98–1.64)	0.07	1.45 (1.05–2.01)	0.024	1.02 (0.74–1.41)	0.91
≥5 glasses/week	Reference	1.46 (1.20–1.79)	<0.001	1.18 (0.88–1.59)	0.27	1.00 (0.77–1.29)	0.98
d							
Spirits drinkers							
Non-drinkers	Reference	Reference		Reference		Reference	
1–2 glasses/week	Reference	1.13 (0.93–1.37)	0.23	1.27 (0.99–1.64)	0.06	1.11 (0.87–1.41)	0.40
3–4 glasses/week	Reference	1.30 (0.98–1.73)	0.08	1.12 (0.78–1.62)	0.53	1.14 (0.83–1.58)	0.42
≥5 glasses/week	Reference	1.23 (0.96–1.60)	0.11	1.28 (0.93–1.78)	0.13	1.06 (0.81–1.38)	0.68
Fortified wine drinkers							
Non-drinkers	Reference	Reference		Reference		Reference	
1–2 glasses/week	Reference	1.14 (0.86–1.53)	0.37	1.15 (0.80–1.65)	0.45	1.13 (0.79–1.62)	0.50
3–4 glasses/week	Reference	1.11 (0.57–2.14)	0.76	0.85 (0.38–1.93)	0.71	0.73 (0.31–1.71)	0.47
≥5 glasses/week	Reference	1.75 (0.89–3.46)	0.11	1.06 (0.39–2.84)	0.92	0.95 (0.41–2.18)	0.90

*Logistic regression analysis adjusted for age, sex, education, ethnicity, employment, overall health rating, smoking, Townsend, BMI, usually walking pace, sleep duration, getting up easily in the morning, nap during the day, daytime dozing/sleeping, sleeplessness, snoring, cardiovascular diseases, diabetes, respiratory disease, cancer, frequency of alcohol intake, red wine, champagne plus white wine, beer and cider, spirits, and fortified wine*.

**Table 9:** Odds ratios and 95% CIs for the interaction effect between beer and cider and the risk of IBD.

	**Beer and cider drinkers**						
	**Non-drinkers**	**1–2 glasses/week**	***P***	**3–4 glasses/week**	***p***	**≥5 glasses/week**	***p***
Overall health rating							
Excellent or good	Reference	Reference		Reference		Reference	
Fair or poor	Reference	0.92 (0.79–1.07)	0.29	0.81 (0.66–0.99)	0.040	0.78 (0.67–0.90)	0.001
Alcohol consumption, dosage							
Never drinker, previous–drinkers or special occasions only	Reference	Reference		Reference		Reference	
Within guidelines	Reference	N/A		N/A		N/A	
Above guidelines	Reference	0.90 (0.75–1.08)	0.25	0.82 (0.64–1.04)	0.10	0.95 (0.72–1.25)	0.73
Double above the guidelines or more	Reference	1.22 (0.98–1.53)	0.08	1.01 (0.77–1.33)	0.95	1.12 (0.85–1.48)	0.43
Spirits drinkers							
Non-drinkers	Reference	Reference		Reference		Reference	
1–2 glasses/week	Reference	1.09 (0.89–1.33)	0.41	1.35 (1.04–1.76)	0.023	0.97 (0.77–1.22)	0.79
3–4 glasses/week	Reference	1.40 (1.04–1.89)	0.026	1.40 (0.98–2.00)	0.06	1.07 (0.79–1.45)	0.64
≥5 glasses/week	Reference	1.46 (1.14–1.88)	0.003	1.11 (0.80–1.55)	0.53	0.93 (0.73–1.18)	0.55
Fortified wine drinkers							
Non-drinkers	Reference	Reference		Reference		Reference	
1–2 glasses/week	Reference	1.12 (0.84–1.48)	0.44	0.90 (0.57–1.40)	0.63	0.89 (0.59–1.33)	0.57
3–4 glasses/week	Reference	1.67 (0.91–3.07)	0.10	0.55 (0.13–2.29)	0.41	0.38 (0.09–1.60)	0.19
≥5 glasses/week	Reference	0.71 (0.30–1.71)	0.45	0.83 (0.25–2.73)	0.76	0.57 (0.20–1.63)	0.30

*Logistic regression analysis adjusted for age, sex, education, ethnicity, employment, overall health rating, smoking, Townsend, BMI, usually walking pace, sleep duration, getting up easily in the morning, nap during the day, daytime dozing/sleeping, sleeplessness, snoring, cardiovascular diseases, diabetes, respiratory disease, cancer, frequency of alcohol intake, red wine, champagne plus white wine, beer and cider, spirits, and fortified wine*.

**Table 10:** Odds ratios and 95% CIs for the interaction effect between spirits and risk of IBD.

	**Spirits drinkers**						
	**Non-drinkers**	**1–2 glasses/week**	***p***	**3–4 glasses/week**	***p***	**≥5 glasses/week**	***p***
Overall health rating							
Excellent or good	Reference	Reference		Reference		Reference	
Fair or poor	Reference	0.97 (0.82–1.13)	0.67	0.88 (0.70–1.10)	0.26	0.88 (0.73–1.06)	0.17
Alcohol consumption, dosage							
Never drinker, previous–drinkers or special occasions only	Reference	Reference		Reference		Reference	
Within guidelines	Reference	N/A		N/A		N/A	
Above guidelines	Reference	0.94 (0.78–1.13)	0.53	0.91 (0.69–1.20)	0.51	0.87 (0.67–1.12)	0.27
Double above the guidelines or more	Reference	0.77 (0.61–0.97)	0.024	1.00 (0.75–1.34)	0.98	0.67 (0.52–0.87)	0.002
Fortified wine drinkers							
Non-drinkers	Reference	Reference		Reference		Reference	
1–2 glasses/week	Reference	1.01 (0.76–1.32)	0.97	0.77 (0.49–1.22)	0.27	0.91 (0.60–1.38)	0.67
3–4 glasses/week	Reference	1.62 (0.76–3.45)	0.21	2.01 (0.94–4.30)	0.07	2.24 (1.07–4.69)	0.032
≥5 glasses/week	Reference	0.99 (0.43–2.26)	0.98	0.29 (0.04–2.13)	0.22	0.67 (0.29–1.53)	0.34

*Logistic regression analysis adjusted for age, sex, education, ethnicity, employment, overall health rating, smoking, Townsend, BMI, usually walking pace, sleep duration, getting up easily in the morning, nap during the day, daytime dozing/sleeping, sleeplessness, snoring, cardiovascular diseases, diabetes, respiratory disease, cancer, frequency of alcohol intake, red wine, champagne plus white wine, beer and cider, spirits, and fortified wine*.

**Table 11:** Odds ratios and 95% CIs for the interaction effect between fortified wine and risk of IBD.

	**Fortified wine drinkers**						
	**Non-drinkers**	**1–2 glasses/week**		**3–4 glasses/week**		**≥5 glasses/week**	
Overall health rating							
Excellent or good	Reference	Reference		Reference		Reference	
Fair or poor	Reference	1.11 (0.87–1.41)	0.41	0.99 (0.56–1.75)	0.98	0.59 (0.32–1.11)	0.10
Alcohol consumption, dosage							
Never drinker, previous–drinkers or special occasions only	Reference	Reference		Reference		Reference	
Within guidelines	Reference	N/A		N/A		N/A	
Above guidelines	Reference	1.09 (0.83–1.43)	0.55	0.80 (0.41–1.53)	0.49	3.01 (0.40–22.51)	0.28
Double above the guidelines or more	Reference	0.98 (0.71–1.34)	0.88	0.67 (0.32–1.40)	0.29	2.51 (0.34–18.62)	0.37

*Logistic regression analysis adjusted for age, sex, education, ethnicity, employment, overall health rating, smoking, Townsend, BMI, usually walking pace, sleep duration, getting up easily in the morning, nap during the day, daytime dozing/sleeping, sleeplessness, snoring, cardiovascular diseases, diabetes, respiratory disease, cancer, frequency of alcohol intake, red wine, champagne plus white wine, beer and cider, spirits, and fortified wine*.

### Non-linear associations of alcoholic beverages with the amount of alcohol consumption on IBD risk

**Figure 1:**
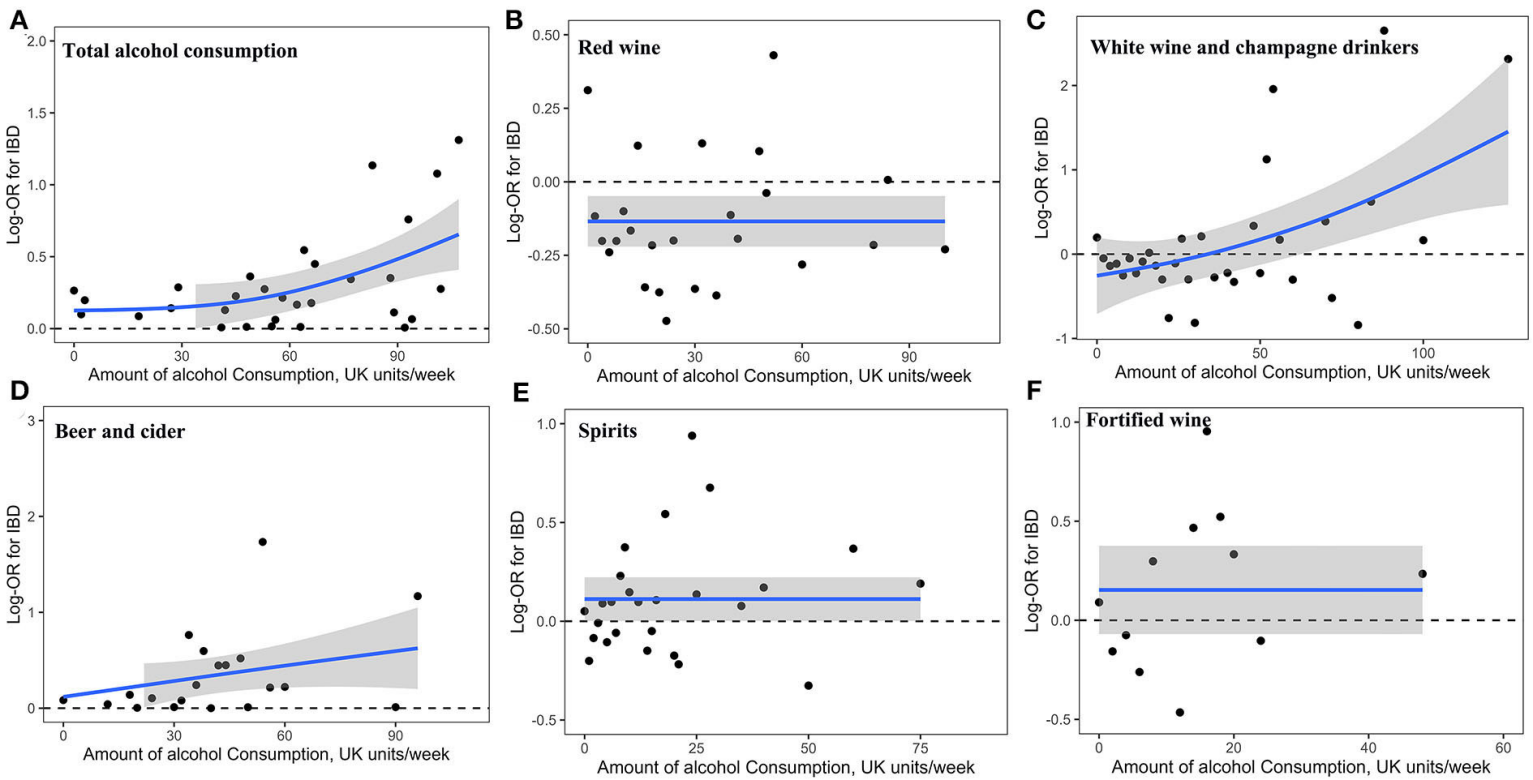
Association of alcohol consumption with risk of IBD. Linear and non-linear associations of the amount of total alcohol consumption **(A)** and that of alcoholic beverages (**B**, red wine; **C** white wine and champagne; **D**, beer and cider; **E**, spirits; **F**, fortified wine) with risk of IBD.

The dose-response associations between the amount of alcohol consumption and alcoholic beverages on IBD risk are shown in Figures 1A–F. The total amount of alcohol consumption (Figure 1A) and white wine and champagne (Figure 1C) showed a curvilinear J-shaped correlation with the risk of IBD, respectively. Red wine, beer and cider, spirits, and fortified wine showed a linear correlation with IBD risk, respectively (Figures 1B,D–F), where alcohol consumers had an increased risk with a greater number of consumptions of beer and cider, and the IBD risk was lower among red consumers across the whole dose cycle.

## Discussion

In this prospective study of a large-sized UK Biobank cohort, we documented four novel findings. First, red wine was identified as an independent protective factor for IBD. It played the protective effect on those subjects who consumed alcohol above or double above the guidelines and consumed alcohol both at high and low frequencies. Second, 1–2 glasses/week of white wine and champagne was identified as an independent risk factor for IBD, especially among those subjects who consumed alcohol double above the guidelines and who consumed alcohol at a high frequency. Third, consumption of ≥5 glasses/week of spirits at a low frequency and consumption of beer and cider double above the guidelines are associated with increased risk of IBD. Fourth, the dose-response associations showed an increased risk of IBD with more number of alcohol consumption. This association can be found in white wine and champagne, and beer and cider. However, red wine is still at a low risk across the whole dose cycle. Therefore, the IBD risk appears to vary across consumption of different subtypes, frequencies, and number of alcoholic beverages. Especially, our study suggests that the association between alcoholic beverages and the risk of IBD is not affected by overall health rating.

Our study found that alcohol intake was associated with a higher risk of IBD. The protective association of low-to-moderate alcohol consumption with lower risk of cataract (23), lower risk of myocardial infarction, (20) and better cognitive function (24) have been widely reported. Recent studies found that chronic alcoholism was not a risk or protective factor of IBD (25, 26). However, studies have shown that long-term alcohol abuse and acute binge drinking are associated with immunosuppression and increased susceptibility to both bacterial and viral infections (27, 28). The widely held view of the health benefits of alcohol needs revising, and Collaborators proposed that the safest level of drinking is none (6). Our results support this view. In our study, the total number of alcohol consumption showed a curvilinear J-shaped correlation with IBD risk, and the recommended intake of alcohol is also none. This level conflicts with most health guidelines, which espouse health benefits associated with low-to-moderate alcohol consumption. However, subgroup analyses for alcoholic beverages reported a new finding, which shows that red wine is safe to drink throughout the whole dose cycle as it was associated with a lower risk of IBD. Therefore, it's worth noting that, although our findings suggest that alcohol intake is not recommended because it was associated with a higher risk of IBD, there is no direct evidence that red wine is not recommended for IBD high-risk population.

The most striking and interesting finding was that consumption of red wine was associated with a lower chance of developing IBD. More and more research are trying to explore the potentially beneficial properties of wine or its components on IBD, and studies have shown that the beneficial properties of wine are attributed to their polyphenolic content, but are not independent of the presence of alcohol (29, 30). Polyphenols are present in varying degrees in different subtypes of alcoholic beverages, particularly in red wine which has the highest concentrations of phenolic compounds (31) because all grape parts are used during the winemaking process of red wine (32). Red wine provides additional benefits compared to other subtypes of alcoholic beverages probably due to its higher polyphenolic content, by decreasing blood pressure, improving endothelial function, reducing inflammation and cell adhesion, inhibiting the oxidation of low-density lipoprotein particles, and other favorable effects on the cellular redox state, inhibiting platelet aggregation, and activating proteins that prevent cell death (31). Polyphenols comprised several chemical compounds that are generally classified into flavonoids and non-flavonoids (33), which are considered the main bioactive components in wine that positively affect health (31, 34). The beneficial effect included antioxidant, anti-inflammatory, anti-cancer, and anti-microbial (35). As known, polyphenols could inhibit the effects of several types of viruses, including the Epstein–Barr virus (36, 37), herpes simplex virus (38), enterovirus (39, 40), COVID-19 (18), influenza virus (41), and other viruses causing respiratory tract-related infections (42, 43). Many of the phenolic compounds in wine have low bioavailability, and hence, reach low concentrations in the bloodstream, while their high content present in the gut can produce a more significant effect on enterocytes and the bacterial flora (44, 45). Therefore, these findings support the notion of the strong beneficial properties of red wine against developing IBD risk.

Our study suggests that consumption of some alcoholic beverages were associated with higher risks of developing IBD. We discovered risk factors of low frequency of ≥5 glasses/week spirit, high frequency and double above dose of the guidelines of 1–2 glasses/week of white wine and champagne, and double above dose of the guidelines of beer and cider for developing IBD. Sanja Radonjić et al. have reported the differences between wine and beer in the presence and the concentrations of phenolic substances (33). Furthermore, spirits had the highest alcohol concentration and the lowest polyphenolic concentration. These findings have shown the differences between these alcoholic beverages and red wine. Evidence have shown that red wine extract and wine digested fluids played a protective effect on the cellular barrier (46, 47) and led to increased intestinal permeability (48). However, this is in stark contrast to the evidence by Asai et al. that a low and acute dose of ethanol leads to apoptotic cell death in confluent Caco-2 cells and, therefore, impairs intestinal barrier function (49). Therefore, it is probable that the polyphenolic content per se has a positive effect on intestinal permeability, while the alcoholic content potentially negates this effect (17). These findings may suggest that the specific class of polyphenolic constituents may be responsible for the beneficial effect of alcoholic beverages on IBD, but not the alcohol concentration.

The major strengths of this study are the prospective and comprehensive study design, large UK population-based cohort, dose-response associations of alcohol consumption with IBD risk, and a focus on the association of different subtypes of alcoholic beverages with IBD risk. However, there are several limitations that should be addressed. First, subjects in the UK Biobank have a restricted age range, and therefore our data could not represent the whole population. Second, the data on alcohol drinking habits were derived from baseline, and we did not know about potential changes from baseline to outcome end-point. Third, recruiting heavy drinkers to test different alcoholic beverages for dose-response analyses is difficult. Fourth, although we adjusted for a wide range of potential confounders and did a series of rigorous statistical analyses, residual confounding factors may still exist, especially the interaction between different alcoholic beverages. Fifth, a relatively small number of IBD cases that only consumed one alcoholic beverage were included, which may reduce its reliability. Sixth, past trauma or stressful events may be a cause of alcohol intake, future studies should adjust for the effect of past trauma or stressful events.

## Conclusions

In conclusion, our study suggests that the IBD risk appears to vary across different frequencies, amounts, and subtypes of alcoholic beverages. Overall, alcohol intake is not recommended because it was associated with a higher risk of IBD, and the safe level of drinking appears to be none. A focus on the association of different frequencies, amounts, and subtypes of alcoholic beverages with the risk of IBD provided an important addition to the existing research on alcohol intake and IBD risk. We found that consumption of red wine may reduce the risk of IBD, while high frequency and high dose of white wine and champagne, low frequency and acute dose of spirit, and high dose of beer and cider appear to increase the risk of IBD. Public health guidance should focus on reducing the risk of IBD by advocating healthy lifestyle habits and preferential policies among consumers.

## Data availability statement

The original contributions presented in the study are included in the article/supplementary materials, further inquiries can be directed to the corresponding author/s.

## Ethical approval

Ethical approval of the UK Biobank was obtained from the National Health System Northwest Multicenter Research Ethics Committee (REC reference: 16/NW/0274). The patients/participants provided their written informed consent to participate in this study.

## Author contributions

B-XL, CZ, X-JD, and YC had the idea for and designed this study. X-JD and B-XL had full access to all the data in this study, take responsibility for the integrity of the data and the accuracy of the data analysis, and drafted the paper. CZ, X-JD, and YC critically revised the manuscript for important intellectual content and gave final approval for the version to be published. JY, CZ, and YC take responsibility for double check of the data analysis. All authors contributed to the article and approved the submitted version.

## Funding

This work was supported by grants from the National Natural Science Foundation of China (Grant No. 82060448) and Sanming Project of Medicine in Shenzhen (Grant No. SZSM201812052). This research has been conducted using the UK Biobank Resource under Application Number 75732 and Basket 2013245.

## Conflict of interest

The authors declare that the research was conducted in the absence of any commercial or financial relationships that could be construed as a potential conflict of interest.

## Publisher's note

All claims expressed in this article are solely those of the authors and do not necessarily represent those of their affiliated organizations, or those of the publisher, the editors and the reviewers. Any product that may be evaluated in this article, or claim that may be made by its manufacturer, is not guaranteed or endorsed by the publisher.
